# Random Amplified Polymorphic Markers as Indicator for Genetic Conservation Program in Iranian Pheasant (*Phasianus colchicus*)

**DOI:** 10.1100/2012/640381

**Published:** 2012-05-02

**Authors:** Ghorban Elyasi Zarringhabaie, Arash Javanmard, Ommolbanin Pirahary

**Affiliations:** ^1^Department of Animal Science, East Azarbaijan Research Center for Agriculture and Natural Resources, Tabriz, Iran; ^2^Department of Genomics, Agricultural Biotechnology Research Institute for Northwest and West of Iran, Tabriz, Iran; ^3^Animal Science Section, East Azerbaijan Jahad-e-Keshavarzi Organization, Tabriz, Iran

## Abstract

The objective of present study was identification of genetic similarity between wild Iran and captive Azerbaijan Pheasant using PCR-RAPD markers. For this purpose, in overall, 28 birds were taken for DNA extraction and subsequently 15 arbitrary primers were applied for PCR-RAPD technique. After electrophoresis, five primers exhibited sufficient variability which yielded overall 65 distinct bands, 59 polymorphic bands, for detalis, range of number of bands per primer was 10 to 14, and produced size varied between 200 to 1500 bp. Highest and lowest polymorphic primers were OPC5, OPC16 (100%) and OPC15 (81%), respectively. Result of genetic variation between two groups was accounted as nonsignificant (8.12%) of the overall variation. According to our expectation the wild Iranian birds showed higher genetic diversity value than the Azerbaijan captive birds. As general conclusion, two pheasant populations have almost same genetic origin and probably are subpopulations of one population. The data reported herein could open the opportunity to search for suitable conservation strategy to improve richness of Iran biodiversity and present study here was the first report that might have significant impact on the breeding and conservation program of Iranian pheasant gene pool. Analyses using more regions, more birds, and more DNA markers will be useful to confirm or to reject these findings.

## 1. Introduction

Pheasants are one of the most endangered species of birds in the world [1] which appeared on the most recent list of endangered species list [2]. Generally, pheasants species refer to any member of the subfamily of Phasianidae in the order Galliformes. There are 35 species of pheasant in 11 genera [3]. The pheasants are Asian bird in their native distributions, with the single exception of the peafowl, which is endemic to the central Africa [4]. The best known is the common pheasant, which is widespread throughout the world in introduced feral populations and in farm operations. It is native to Russia and has been widely introduced elsewhere as a game bird. 

Iranian pheasant is one of the unique and unmixed species of world which is on the edge of extinction now. These species are on the list of the world's threatened species. Therefore, conservation program aims to preserve the genetic distinctiveness of the species in the north of Iran [5]. The belief is that dwindling wild populations of any such species can be supplemented (i.e., restocked) or reintroduced after extinction from the species' native range by releasing individuals from ex situ populations back into the wild. Pheasant propagation programs and subsequence releasing of young pheasant to their habitat could be possible practical way to increase number of Iranian pheasant conservation [5].

Azerbaijan country as neighbor country of Iran has many captive pheasant farms and could be as good potential for importing eggs or live birds for pheasant propagation programs. There is several seasonal immigrations between borders of these counties reported by environmental protection organization of Iran. There is limited scientific information about genetic structure of pheasant in Iran. Identification of genetic similarity between two countries could provide scientific evidence of genetic relatedness between two counties pheasant population.

Conservation attempts for wild birds have been limited; management practices are based almost solely on controlled burning, leaving areas of varying postfire age to maintain optimal habitat availability. The analysis of genetic variability is an essential ingredient for conservation programs, and the approach must be based on a combination of phenotypic and genetic data [6].

These indigenous birds such as pheasant are now subjected to fast genetic degradation and dilution because of unplanned conservation and introduction of exotic germplasm, so evaluation of DNA marker is prerequired before designing of any strategy for conservation. The use of molecular markers can aid in the choice of breeds and populations to be conserved, when there is a shortage of resources, as well as the estimation of genetic variability of species breeds and populations [7].

Random amplified polymorphic DNA (RAPD) is a useful approach to assessing genetic variation for conservation of wild populations; it is based on PCR amplification of genomic DNA with arbitrary nucleotide sequence primers. The RAPD marker can detect high levels of DNA polymorphism and can produce fine genetic markers [8, 9]. This method is simple and quick to perform when there is no prior knowledge about the genetic make-up of the organism [10]. Nevertheless, RAPD analysis has some limitations. It shows dominant inheritance, and marker/marker homozygotes cannot be distinguished from marker/null heterozygote [8].

The objective of present study was identification of genetic similarity between Iran and Azerbaijan Pheasant birds using PCR-RAPD Markers.

## 2. Material and Methods

### 2.1. Birds

Birds were taken from Arasbaran region (Jolfa, Karanlu) one of the most important border areas of Iran with a varying altitude from 256 m in the vicinity of Aras River to 2896 m and covering an area of 78560 hectares and also commercial farm of Bardeh city near Baku in Azerbaijan country. In overall, 28 birds (Iranian wild pheasant *n* = 16 (coded: 1–16) captives Azerbaijan Pheasant *n* = 12 (coded: 17–28)) were used for present study.

### 2.2. DNA Extraction

Blood samples for DNA genotyping were collected from hunted and live birds using feathertrap and stored at −20°C for few weeks or −70°C up to several months. DNA in tissues or feathers from museum skins was used for DNA extraction as well. DNA extraction was done using commercial kit [11]. Relative purity of the DNA was determined using a spectrophotometer based on absorbance at 260 and 280 nm. The sequences of the primers and the annealing temperatures used were as previously reported studies [12].  Table 1 shows sequence, GC content, and melting temperature of primers.

### 2.3. PCR Protocol

PCR was carried out in 25 *μ*L volumes comprising of 1.5 mM MgCl_2_, 0.2 mM dNTP, 0.01 mM of primer (Fermentas), 50 ng of genomic DNA, and 1 U Taq DNA polymerase (Promega). The PCR-RAPD protocol included initial denaturation for 3 min at 94°C, followed by 34 cycles of denaturation for 45 s at 94°C, annealing for 45 s at 37°C, extension for 1 min at 72°C, and a final extension at 72°C for 10 min. PCR products were electrophoresed at 85 V for 45 min in 2.5% agarose gels and viewed under UV light. The sizes of alleles were determined in relation to a 100 bp DNA size standard (Fermentas) using a computer software BIO 1D++. 

### 2.4. Statistical Analysis

Produced loci (band), number of polymorphic band, gene diversity Shannon's information, and polymorphic percentage (%) were calculated with POPGENE 3.1 [13]. The analyses of molecular variance (AMOVA), which estimated population differentiation, were carried out by Arlequin ver. 3.1 software [14]. Principle component analyses were graphed using PAST and NTSYS softwares.

## 3. Results

Five primers among total 15 random primers exhibited sufficient variability for studied populations. To score the band pattern, we assumed that one band corresponded to one locus. After the duplication tests, it was concluded that the RAPD bands acquired in this study are reproducible. The number of polymorphic bands varied from 10 to 14, with a range of 200–1500 bp (Table 2).

**Figure 1:**
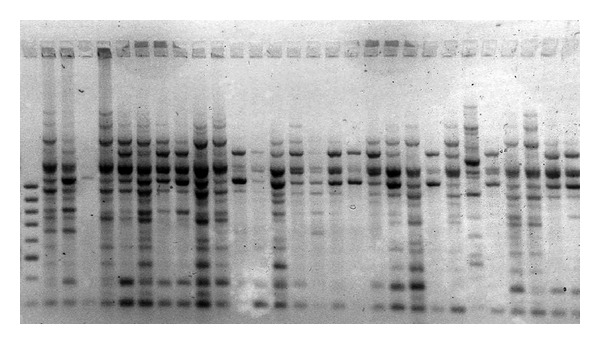
Amplified PCR product using OPC-02 primer.

**Figure 2:**
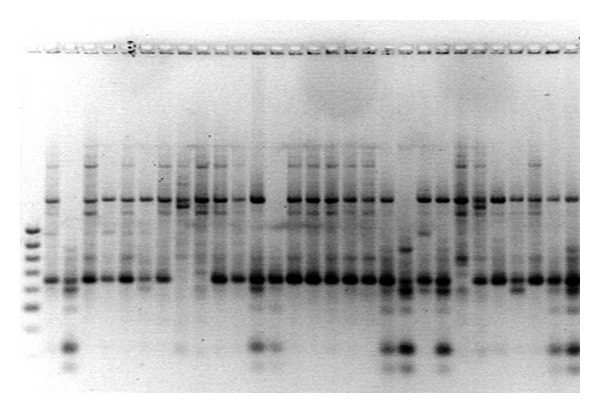
Amplified PCR product using OPC-05 primer.

**Figure 3:**
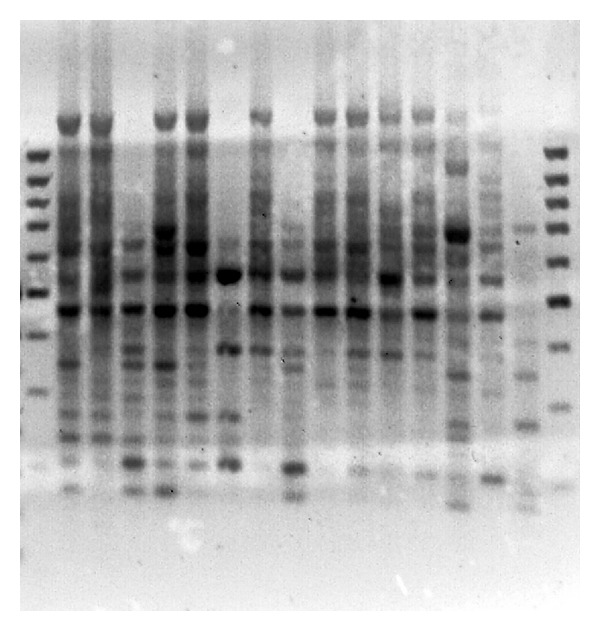
Amplified PCR product using OPC-08 primer.

**Figure 4:**
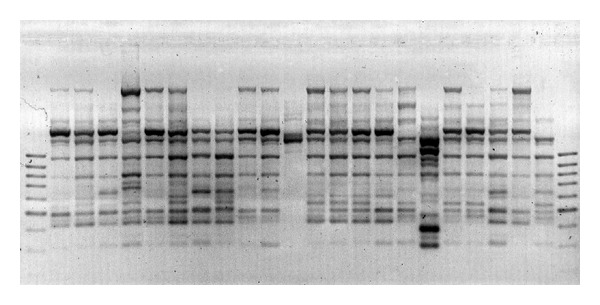
Amplified PCR product using OPC-15 primer.

**Figure 5:**
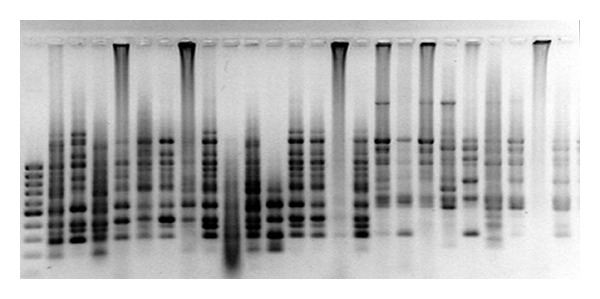
Amplified PCR product using OPC-16 primer.

The five primers yielded 65 distinct bands, 59 of which were polymorphic (Figures 1, 2, 3, 4 and 5). Highest and lowest polymorphism were OPC5, OPC16 (100%) and OPC15 (81%), respectively (Table 2). AMOVA also revealed that genetic variation between the two countries accounted for nonsignificant 8.12% of the total variation (Table 3).

**Figure 6:**
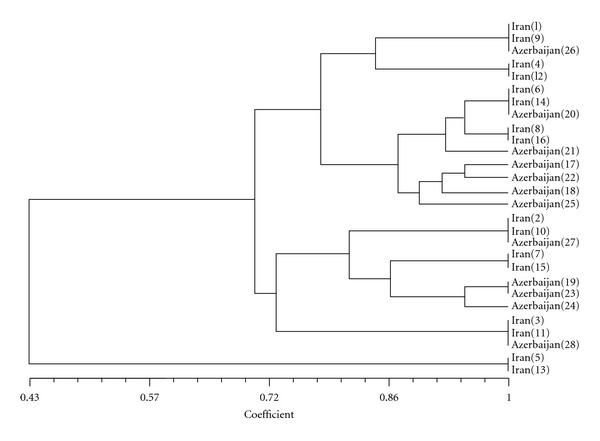
Cluster analysis of PCR-RAPD result using NTSYS software.

**Figure 7:**
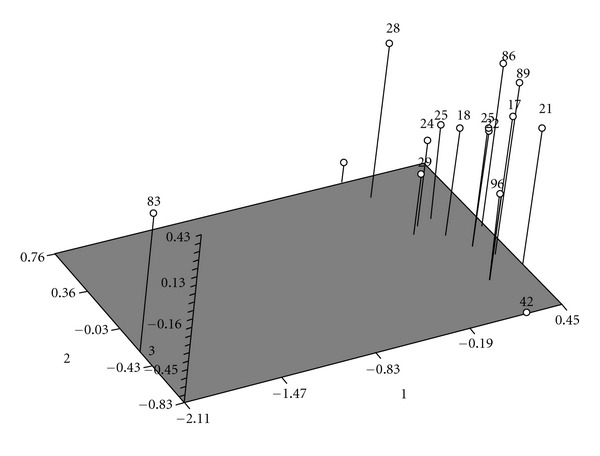
PCA analysis for classification of birds using NTSYS software.

Estimation of polymorphic loci, intrapopulation similarity indices suggested 96.5 percent similarity between two populations. It concluded that two investigated pheasant populations have same genetic origin and probably we can assume them as subpopulations of one population (Figures 6 and 7).

## 4. Discussion 

Genetic diversity is a major issue of conservation biology recognized by the IUCN [15]. Within a population it reflects the evolutionary potential to adapt to novel environmental changes.****

 Therefore, during the past few years, the genetic diversity of many threatened mammals, birds, fish, insects, and plants has been investigated [15]. Recently, concerns have become focused on the issue of the genetic integrity and conservation status of free ranging populations of pheasant. 

The common pheasant (*Phasianus colchicus) *occurs widely in forested regions in northern Iran. Management of populations for sport hunting has centred on the south Caspian region, where special management areas have been set aside and stocks manipulated. Hunting of this bird is illegal in all seasons and some of its important residences such as the national park of Golestan, the preserved area of central Alborz, Miankaleh, Samkandeh, and Arasbaran are among the preserved areas. Unfortunately, the illegal hunting and destruction of its environment have exposed this species to a serious danger. 

From the analysis conducted in present study, it is clear that the wild birds have higher genetic diversity than the captive individuals. Therefore genetic diversity of wild Iranian pheasant was higher than Azerbaijan captive birds. There is no doubt that the reduction of genetic diversity has the tendency to compromise the ability of the populations to evolve to cope with novel environmental changes and reduces their chances of long-term existence. 

A good understanding of population genetic structure is critical to the design of an effective conservation programme for this species. Once genetic resources have been identified and characterized, two basic conservation activities follow, which may be defined as in situ and ex situ. Molecular markers provide important measures for population genetic structures and geographic differentiations, which are especially widely used in analyses of intraspecific phylogeographical patterns. 

The belief is that dwindling wild populations of any such species can be supplemented (i.e., re-stocked) or re-introduced after extinction from the species' native range by releasing individuals from ex situ populations back into the wild. Pheasant propagation programs and subsequence releasing of young pheasant to their habitat could be possible practical way to increase number of Iranian pheasant conservation. Result of this study supported the hypothesis that captives Azerbaijan pheasant could be good resource for richness of genetic resource of Iran. It concluded that two investigated pheasant populations have same genetic origin and probably we can assume them as subpopulations of one population. 

These results demonstrate high genetic similarity of Iranian and Azerbaijan pheasant using RAPD markers. The reason for a difference in the polymorphism level for present populations and other similar species seems particularly due to different genetic makeup, species adaptation, natural selection, history of birds, migration, and mutation, even more by technical staff, which may influence the analysis. The data reported here open the opportunity to search for proper conservation strategy forincreasing population of pheasant in Iran. Analyses using more birds and more DNA markers will be useful to confirm or to reject these findings. 

## Figures and Tables

**Table 1:** Sequence, GC content, and melting temperature of primers.

Primer ID	Sequence (5′→3′)	GC	Tm
OPA04	GGCACGCGTT	70	34
OPA07	GAAACGGGTG	60	32
OPC02	GTGAGGCGTC	70	34
OPC05	GATGACCGCC	70	34
OPC06	ATGCCCCTGT	60	32
OPC07	AAAGCTGCGG	60	32
OPC08	TGGACCGGTG	70	34
OPC10	CTGCTCGAGT	60	32
OPC11	TGGACCGGTG	70	34
OPC15	TGAGCGGACA	60	32
OPC16	CACACTCCAG	60	32
OPE02	AGGCCCCTGT	70	34
OPE05	ATGCCCCTGT	70	34
OPM10	TCTGGCGCAC	70	34
OPP11	AACGCGTCGG	70	34

**Table 2:** Statistics of polymorphic primers for investigated populations.

Primer	Amplified bands	Polymorphic bands	Band size (bp)	Polymorphism
OPC02	16	14	100–1450	87.5
OPC05	13	13	100–1500	100
OPC08	12	10	100–1380	83
OPC15	16	13	200–1500	81
OPC16	11	11	100–1300	100

Total	68	59		86.7

**Table 3:** Result of AMOVA for two groups of pheasants.

Source of variation	Variance Component	% Variance	*P* value
Between populations	0.84 V_a_	8.32	ns
Within populations	9.2 V_b_	91.88	<0.01

## Abbreviation

RAPD: Randomly amplified polymorphic DNA
PCR: Polymerase chain reaction
bp: Base pair
dNTP: Dinucleotide triphosphate
MgCl_2_: Magnesium chloride
ng: Nano-gram
1X: One time
°C: Centigrade Celsius.
